# Fis Is Essential for Capsule Production in *Pasteurella multocida* and Regulates Expression of Other Important Virulence Factors

**DOI:** 10.1371/journal.ppat.1000750

**Published:** 2010-02-05

**Authors:** Jason A. Steen, Jennifer A. Steen, Paul Harrison, Torsten Seemann, Ian Wilkie, Marina Harper, Ben Adler, John D. Boyce

**Affiliations:** 1 Australian Research Council Centre of Excellence in Structural and Functional Microbial Genomics, Monash University, Clayton, Victoria, Australia; 2 Victorian Bioinformatics Consortium, Monash University, Clayton, Victoria, Australia; 3 Veterinary Pathology and Anatomy, University of Queensland, St Lucia, Queensland, Australia; 4 Department of Microbiology, Monash University, Clayton, Victoria, Australia; Dartmouth Medical School, United States of America

## Abstract

*P. multocida* is the causative agent of a wide range of diseases of animals, including fowl cholera in poultry and wild birds. Fowl cholera isolates of *P. multocida* generally express a capsular polysaccharide composed of hyaluronic acid. There have been reports of spontaneous capsule loss in *P. multocida*, but the mechanism by which this occurs has not been determined. In this study, we identified three independent strains that had spontaneously lost the ability to produce capsular polysaccharide. Quantitative RT-PCR showed that these strains had significantly reduced transcription of the capsule biosynthetic genes, but DNA sequence analysis identified no mutations within the capsule biosynthetic locus. However, whole-genome sequencing of paired capsulated and acapsular strains identified a single point mutation within the *fis* gene in the acapsular strain. Sequencing of *fis* from two independently derived spontaneous acapsular strains showed that each contained a mutation within *fis*. Complementation of these strains with an intact copy of *fis*, predicted to encode a transcriptional regulator, returned capsule expression to all strains. Therefore, expression of a functional Fis protein is essential for capsule expression in *P. multocida*. DNA microarray analysis of one of the spontaneous *fis* mutants identified approximately 30 genes as down-regulated in the mutant, including *pfhB_2*, which encodes a filamentous hemagglutinin, a known *P. multocida* virulence factor, and *plpE*, which encodes the cross protective surface antigen PlpE. Therefore these experiments define for the first time a mechanism for spontaneous capsule loss in *P. multocida* and identify Fis as a critical regulator of capsule expression. Furthermore, Fis is involved in the regulation of a range of other *P. multocida* genes including important virulence factors.

## Author Summary

*Pasteurella multocida* is an animal pathogen of worldwide economic significance. It causes fowl cholera in wild birds and poultry, hemorrhagic septicemia in ungulates, and atrophic rhinitis in swine. The major virulence factor in fowl cholera-causing isolates is the polysaccharide capsule, which is composed of hyaluronic acid. Although there have been reports of spontaneous capsule loss in some strains, to date there has been no systematic investigation into the molecular mechanisms of this phenomenon. In this study, we describe for the first time the underlying transcriptional mechanisms required for the expression of capsule in *P. multocida*, and identify a transcriptional regulator required for capsule production.

## Introduction

*Pasteurella multocida* is an important veterinary pathogen of worldwide economic significance; it is the causative agent of a range of diseases, including fowl cholera in poultry, hemorrhagic septicemia in ungulates and atrophic rhinitis in swine. *P. multocida* is a heterogeneous species, and is generally classified into five capsular serogroups (A, B, D, E and F) and 16 somatic LPS serotypes (1–16) [1]. Each serogroup produces a distinct capsular polysaccharide, with serogroups A, D and F producing capsules composed of hyaluronic acid (HA) [2], heparin and chondroitin [3], respectively. The structures of the serogroup B and E capsules are not known, although preliminary compositional analysis suggests that these capsules have a more complex structure than those produced by serogroups A, D and F [4]. The genes involved in biosynthesis, export and surface attachment of the capsular polysaccharide have been identified for all capsule types [5]–[7]. For strains which express HA, the capsule biosynthetic locus (*cap*) consists of 10 genes; *phyA* and *phyB* are predicted to encode proteins responsible for lipidation of the polysaccharide, *hyaE*, *hyaD*, *hyaC* and *hyaB* encode proteins required for polysaccharide biosynthesis and *hexD*, *hexC*, *hexB* and *hexA* encode proteins responsible for transport of the polysaccharide to the bacterial surface [6].

The *P. multocida* capsule is a major virulence determinant in both serogroup A and B strains. In the serogroup A strain X-73 (A:1), inactivation of the capsule transport gene *hexA* resulted in a mutant strain that was highly attenuated in both mice and chickens, and was more sensitive to the bactericidal activity of chicken serum [8]. Similarly, mutation of the *hexA* orthologue, *cexA*, in the serogroup B strain M1404 also resulted in significant attenuation; the M1404 *cexA* mutant was 4–6 times more sensitive than the parent to phagocytosis by murine macrophages [9].

Spontaneous capsule loss during *in vitro* sub-culture has been described in *P. multocida* [10],[11]. In one study, acapsular variants were derived from capsulated parent strains by repeated laboratory passage (>30 sub-cultures) [12]. Sequence analysis of the *cap* locus in one of these acapsular variants identified two nucleotide changes near the putative promoter region, but the authors did not determine whether these changes were responsible for the observed acapsular phenotype. No further work has been published on the genetic mechanisms of regulation of *P. multocida* capsule production.

Fis is a growth phase-dependent, nucleoid-associated protein which plays a role in the transcriptional regulation of a number of genes in diverse bacterial species (reviewed in [13]). In *Escherischia coli*, Fis is expressed at high levels in actively growing cells (>50 000 molecules per cell in early exponential growth phase), and expression drops to very low levels during stationary phase [14],[15]. In addition to growth phase regulation, levels of Fis are negatively regulated by the stringent response during nutrient starvation [16]. Fis can act as both a positive or negative regulator of transcription and it has both direct and indirect effects on gene transcription. In *E. coli* and *Salmonella*, Fis binds to a degenerate 15-bp consensus sequence GNtYAaWWWtTRaNC, inducing DNA bending, but only a few of the sequences fitting this consensus are high affinity binding sites [17],[18]. Fis is involved in the regulation of genes encoding a wide range of functions, including quorum sensing in *Vibrio cholerae* [19], and certain virulence factors in *Erwinia chrysanthemi* [20], pathogenic *E. coli* [21],[22] and *Salmonella* [23].

In this study, we have characterized three independently isolated spontaneous acapsular derivatives of the *P. multocida* A:1 strain VP161. Whole genome sequencing and DNA microarrays were used to show that the global regulator Fis not only controls the expression of capsule biosynthesis genes in *P. multocida*, but also regulates a number of other genes, including known and putative virulence factors.

## Results

### Identification of spontaneous acapsular *P. multocida* strains

**Figure 1:**
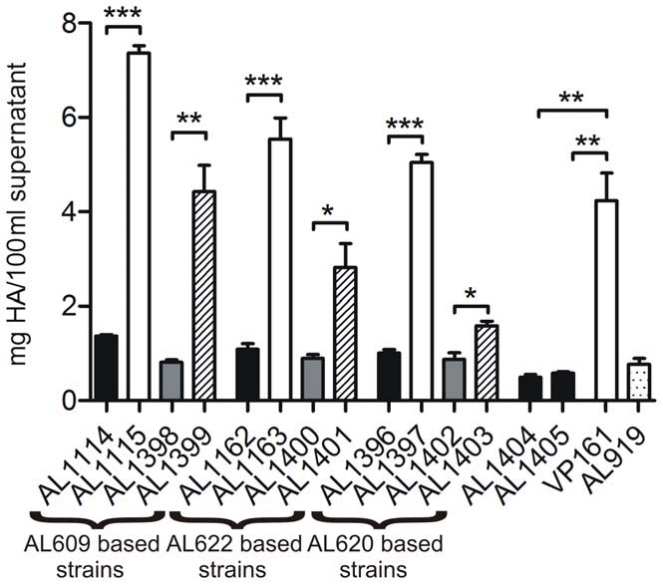
Hyaluronic acid (HA) capsular polysaccharide produced by *P. multocida* strains. The amount of capsular material is shown for paired acapsular (AL1114, AL1162 and AL1396) and capsulated (AL1115, AL1163 and AL1397) variants and the same strains complemented with either empty vector (AL1398, AL1400 and AL1402) or an intact copy of *fis* (AL1399, AL1401 and AL1403). Brackets below the figure indicate strains derived from the same parent strain. Also shown is the amount of HA produced by the TargeTron *fis* mutants AL1404 and AL1405, the wild-type encapsulated parent strain VP161 and the *hyaB* capsule mutant AL919. Results for experimentally related strains are shaded similarly with black bars representing *fis* mutants, white bars representing parent and wild-type strains, hatched bars representing the *fis* complemented strains, gray bars representing strains transformed with vector only and the dotted bar representing the directed *hyaB* mutant. Each bar represents triplicate values ±1 standard error of the mean (SEM) *** p<0.001, ** p<0.01, * p<0.05.

Spontaneous capsule loss has been reported previously in *P. multocida*, and is generally associated with long term *in vitro* passage on laboratory media [10]–[12]. During routine strain maintenance of a signature-tagged mutagenesis library, we identified three independent *P. multocida* strains that presented with both large mucoid and small non-mucoid colonies after recovery from short term (<1 year) −80°C glycerol storage. Re-isolation of either colony type resulted in stable populations with colony morphologies identical to those of their parents, such that AL609, AL622 and AL620 gave rise to the small non-mucoid variants AL1114, AL1162 and AL1396 and to the large mucoid variants AL1115, AL1163 and AL1397, respectively (Table 1). Quantitative HA assays confirmed that all three small, non-mucoid colony variants (AL1114, AL1162 and AL1396) expressed significantly less capsular material than their paired large, mucoid colony variants (AL1115, AL1163 and AL1397) and the parental VP161 strain (Fig. 1). Indeed, the small colony variants expressed similar levels of HA to that expressed by a defined (*hyaB*) acapsular polysaccharide biosynthesis mutant generated by single crossover allelic exchange (AL919; Table 1) (Fig. 1). As each of the paired strains was derived from transposon mutants with different transposon insertion sites (AL609, AL622 and AL620; Table 1), and the transposon was still present at identical positions in both the paired mucoid and non-mucoid derivatives of each type, we concluded that the acapsular phenotype was independent of the initial transposition event.

**Table 1:** Strains and plasmids used in this study.

Strains/plasmid	Genotype and Description	Reference/source
Strains		
*E. coli*		
Sm10 λ *pir*	Strain for propagation of pUA826 and its derivatives.	[69]
AL912	Sm10 λ *pir* containing pAL499	This study
*P. multocida*		
VP161	Wild type *P. multocida* serotype A:1 strain. Capsulated and virulent	[70]
AL435	VP161 carrying a Tn*916* insertion in *pm1417*. Capsulated and virulent. Tet^R^	[57]
AL609	VP161 carrying a Tn*916* insertion in *viaA*	This study
AL620	VP161 carrying a Tn*916* insertion between *galR* and *mglB*	This study
AL622	VP161 carrying a Tn*916* insertion between *thiM* and *pm1263*	This study
AL919	*S*ingle cross-over *hyaB* mutant of AL435. Expresses no polysaccharide capsule	This study
AL1114	Small non-mucoid colony variant of AL609. Expresses no polysaccharide capsule	This study
AL1115	Large mucoid colony variant of AL609. Expresses polysaccharide capsule	This study
AL1162	Small non-mucoid colony variant of AL622. Expresses no polysaccharide capsule	This study
AL1163	Large mucoid colony variant of AL622. Expresses polysaccharide capsule	This study
AL1164	VP161 carrying a Tn*916* insertion in the capsule biosynthesis gene *phyB*. Contains the plasmid pAL597	This study
AL1396	Small non-mucoid colony type derivative of AL620. Expresses no polysaccharide capsule	This study
AL1397	Large mucoid colony type derivative of AL620. Expresses polysaccharide capsule	This study
AL1398	AL1114 containing vector pPBA1100	This study
AL1399	AL1114 containing pAL727	This study
AL1400	AL1162 containing vector pPBA1100	This study
AL1401	AL1162 containing pAL727	This study
AL1402	AL1396 containing vector pPBA1100	This study
AL1403	AL1396 containing pAL727	This study
AL1404	VP161 marker-free *fis* TargeTron mutant generated using pAL706	This study
AL1405	VP161 marker-free *fis* TargeTron mutant generated using pAL708	This study
AL1571	VP161 containing pAL795	This study
AL1572	AL1114 containing pAL795	This study
AL1573	AL1115 containing pAL795	This study
AL1574	VP161 containing pAL796	This study
AL1575	AL1114 containing pAL796	This study
AL1576	AL1115 containing pAL796	This study
AL1577	VP161 containing pAL797	This study
AL1578	AL1114 containing pAL797	This study
AL1579	AL1115 containing pAL797	This study
AL1580	VP161 containing pAL798	This study
AL1581	AL1114 containing pAL798	This study
AL1582	AL1115 containing pAL798	This study
AL1583	VP161 containing pAL799	This study
AL1584	AL1114 containing pAL799	This study
AL1585	AL1115 containing pAL799	This study
AL1586	VP161 containing pMKΩ	This study
AL1587	AL1114 containing pMKΩ	This study
AL1588	AL1115 containing pMKΩ	This study
AL1654	VP161 containing pAL597	This study
AL1655	AL1114 containing pAL597	This study
AL1656	AL1115 containing pAL597	This study
AL1657	VP161 containing pAL596	This study
AL1658	AL1114 containing pAL596	This study
AL1659	AL1115 containing pAL596	This study
Plasmids		
pJIR750ai	Commercial TargeTron vector	Sigma-Aldrich
pAL99	*P. multocida/E. coli* expression vector. Cloned fragments are expressed from the *P. multocida tpiA* promoter	[71]
pAL499	pUA826tpi containing a 720 bp internal fragment of *hyaB*. For single-crossover insertional mutagenesis.	This study
pAL596	pMKΩ containing the 738 bp intergenic region between *phyA* and *hyaE*. Has the *phyA* promoter directing expression of the kanamycin resistance gene.	This study
pAL597	pMKΩ containing the 738 bp intergenic region between *phyA* and *hyaE*. Has the *hyaE* promoter directing expression of the kanamycin resistance gene.	This study
pAL692	*P. multocida*-specific TargeTron vector. Strep^R^Spec^R^	This study
pAL705	*P. multocida*-specific TargeTron vector. Kan^R^	This study
pAL706	pAL705 retargeted to *fis* using BAP5932, 5933, 5934	This study
pAL708	pAL692 retargeted to *fis* using BAP5932, 5933, 5934	This study
pAL727	Fis complementation construct containing a 1415 bp *Bam*HI/*Sal*I fragment containing *fis* and the upstream overlapping ORF *pm1087* cloned in pPBA1100	This study
pAL795	pMKΩ containing the 206 bp region upstream of *pglA*	This study
pAL796	pMKΩ containing the 466 bp region upstream of *pm0998*	This study
pAL797	pMKΩ containing the 411 bp region upstream of *pm1078*	This study
pAL798	pMKΩ containing the 1049 bp region upstream of *lspB_2*	This study
pAL799	pMKΩ containing the 400 bp region upstream of *pm1818*	This study
pPBA1100	*P. multocida-E. coli shuttle vector*	[72]
pMKΩ	*P. multocida* promoter detecting vector	[24]
pUA826tpi	λ *pir* dependent conjugative plasmid for single-crossover mutagenesis in *P. multocida*. A modified derivative of pUA826 containing the *P. multocida tpiA* promoter which allows for the transcription of downstream genes after insertional mutagenesis. Strep^R^, Spec^R^	[57]

### Decreased capsule production in the acapsular strains results from decreased transcription of the *cap* biosynthetic locus

**Figure 2:**
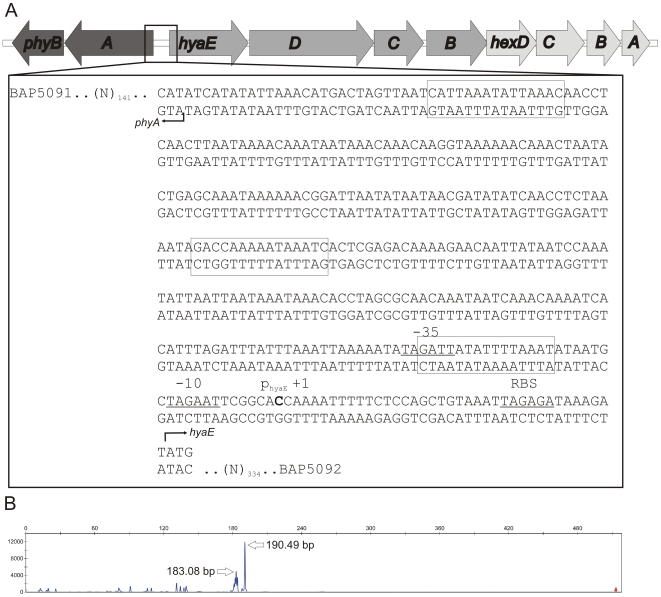
Schematic representation of the *P. multocida* serogroup A capsule biosynthetic locus and identification of the *hyaE* transcriptional start site. The organization of the genes in the capsule biosynthetic locus is shown in panel A and the nucleotide sequence of the intergenic region between *phyA* and *hyaE* is shown in the flyout box. The positions of the HyaE and PhyA translational initiation codons (ATG) are designated by right-angled arrowheads and the identified transcriptional start site for *hyaE* is shown in bold text and labeled +1. Predicted RBS, −10 and −35 sites are underlined, and labeled above the sequence. Predicted Fis binding sites based on the *E. coli* position specific weight matrix are boxed [15]. The length (x-axis) and quantity (y-axis) of *hyaE*-specific primer extension products generated using VP161 total RNA and the primer BAP5476 are shown in panel B. The exact size (in bp) of the major primer extension products are shown beside the peaks.

The genes responsible for HA capsule polysaccharide biosynthesis and transport have been identified previously and are located in a single region of the *P. multocida* chromosome [6] (Fig. 2). In order to investigate whether the loss of capsule production in these spontaneously arising acapsular variants was due to a mutation in the *cap* biosynthetic locus, the nucleotide sequence of the entire 14,935 bp locus of AL1114 and the wild-type parent VP161 was determined. Comparison of the sequences revealed that they were identical; indicating that the acapsular phenotype observed in AL1114 was not due to mutation within the *cap* locus.

**Figure 3:**
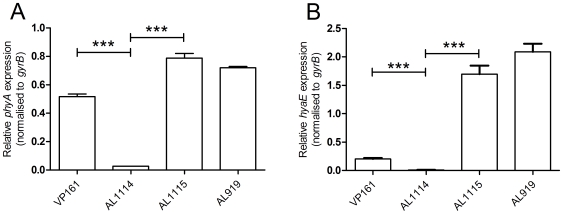
Relative expression of *phyA* and *hyaE* in four *P. multocida* strains. The expression levels of *phyA* (panel A) and *hyaE* (panel B) were determined for the strains VP161 (wild-type), AL1114 (spontaneous acapsular strain), AL1115 (paired capsulated strain) and AL919 (a VP161 *hyaB* insertional mutant) by qRT-PCR and normalized to the level of *gyrB* expression. The average relative expression was determined from three biological replicates and the values shown are the mean ±1 SEM. ** p<0.01, * p<0.05.

As there were no sequence changes observed between these two strains, we investigated transcription across the *cap* locus by quantitative real-time RT-PCR (qRT-PCR). Transcription of the *P. multocida* capsule biosynthetic genes is predicted to initiate from divergent promoters located in the intergenic region between the divergent *phyA* and *hyaE* genes (Fig. 2, [6]). Transcription of *phyA* and *hyaE* was significantly reduced in the acapsular variant AL1114 as compared to both the paired capsulated strain AL1115 and the parent strain VP161 (Fig. 3). Furthermore, a directed *P. multocida hyaB* mutant (AL919, Table 1), showed high levels of transcription across both genes showing that transcription across the locus is not affected by the level of capsular polysaccharide on the cell surface. These data show that the reduced capsule production in the acapsular variant AL1114 was likely a result of reduced transcription across the biosynthetic locus.

To characterize further the transcriptional regulation of the *cap* locus, we identified the position of the *hyaE* transcriptional start site by fluorescent primer extension (Fig. 2). A primer extension product was generated from *P. multocida* VP161 RNA using the primer BAP5476 (Table 2). A 190 bp extension fragment was detected, (Fig. 2B), which identified the transcript start site for *hyaE* as 37 bp upstream of the *hyaE* start codon (Fig. 2). The −10 and −35 regions of the *hyaE* promoter were predicted based on the identified transcript start site (Fig. 2). Repeated attempts failed to detect a primer extension product corresponding to *phyA*.

**Table 2:** Oligonucleotides used in this study.

Oligo	Sequence (5′-3′)^a^	Description
BAP169	GTAAAGGATTGCGATTGC	Reverse primer flanking *hyaB*
BAP276	GAAACCTAAAACTGTTGG	Forward primer flanking *hyaB*
BAP2782	GCCCTACACAAATTGGGAGA	pUA826 specific primer
BAP4399	GAAGAAGTCGACAAGTTGCCGTCATGTGTGTT	*hyaB* internal forward primer for mutagenesis.
BAP4400	GCGTGCGTCGACTTCGCTATCTGCACCGATT	*hyaB* internal reverse primer for mutagenesis
BAP4995	CGACTTCGAGCATCTGGGATA	qRT-PCR of the VP161 *hyaE*
BAP4996	GGTCAAGGTTAGCCCAATAATCTAAT	qRT-PCR of the VP161 *hyaE*
BAP4997	ATGCCGATCATCCGTCAATC	qRT-PCR of the VP161 *phyA*
BAP4998	TGACATCTCACACTGGGTTTGAG	qRT-PCR of the VP161 *phyA*
BAP5091	CATGTTGGATCCTTTTTCCATGTGACAAATGGAGAG	Forward primer for amplification of the intergenic region between *phyA* and *hyaE*
BAP5092	TCGTTTGGATCCGCTTATGAGATTTATTTTTGGCT	Reverse primer for amplification of the intergenic region between *phyA* and *hyaE*
BAP5476	GGTAATACTTTATTAAGCACATGGCCAATT	5′-6-FAM labelled primer for fluorescent primer extension of *hyaE* promoter
BAP5358	GTTCGATATCGCGAAACGATCCTCATCCTG	For amplification of pAL99 vector excluding the kanamycin resistance gene
BAP5359	CCGTGATATCGCTGAAGAGCTTGGCGGC	For amplification of pAL99 vector excluding the kanamycin resistance gene
BAP5360	CGAAGATATCAGACATTATTTGCCGACTACC	For amplification of the *aadA* gene from pUA826
BAP5361	AATGGATATCGCGTATGCGCTCACGCAAC	For amplification of the *aadA* gene from pUA826
BAP5433	CTGGGATATCCTCTGGTAAGGTTGGGAAGC	For amplification of the kanamycin resistance gene from pAL99
BAP5434	CCCAGATATCGCCAAGCTCTTCAGCAATATC	For amplification of the kanamycin resistance gene from pAL99
BAP5540	CGGTAGCGCTGGAAATCG	qRT-PCR of the VP161 *plpE*
BAP5541	AAGAGAATCATTCGTAGCGGTATTAGT	qRT-PCR of the VP161 *plpE*
BAP5826	GAGCATGGATCCCCACCACAATGTCAGCCAC	Reverse primer for amplification of predicted *pm1078* promoter region
BAP5827	CCAACAGGATCCATCCCTTAAACAATATAGCCAAAC	Forward primer for amplification of predicted *pm1078* promoter region
BAP5828	TACACCGGATCCTATTCGTCTAAGAAGCGACTC	Forward Primer for amplification of predicted *pm0998* promoter region
BAP5829	TTATTTGGATCCCAAACCTCGTATAAAATGAATTG	Reverse primer for amplification of predicted *pm0998* promoter region
BAP5832	AACCAAGGATCCGCGCTAAAGTGCGGTTAAATC	Forward primer for amplification of predicted *lspB_2* promoter region
BAP5833	AGTAGCGGATCCACATAATAATAATTTCCCTAATCT	Reverse primer for amplification of predicted *lspB_2* promoter region
BAP5834	ACACTTGGATCCGAATTATAACGCACGCTGTG	Forward primer for amplification of predicted *pglA* promoter region
BAP5835	CATGGTGGATCCGATTTTTAGTCATTTGTTTTTGAG	Reverse primer for amplification of predicted *pglA* promoter region
BAP5838	TGCGGTGGATCCTTTCACATCACCGCTACGT	Reverse primer for amplification of predicted *pm1818* promoter region
BAP5839	CAAATTGGATCCCAATCTGCCCAAGTGCTGC	Forward Primer for amplification of predicted *pm1818* promoter region
BAP5932	AAAAAAGCTTATAATTATCCTTACTAACCGTATCAGTGCGCCCAGATAGGGTG	Fis - 3203|3204s-IBS.
BAP5933	CAGATTGTACAAATGTGGTGATAACAGATAAGTCGTATCAGTTAACTTACCTTTCTTTGT	Fis - 3203|3204s-EBS1d
BAP5934	TGAACGCAAGTTTCTAATTTCGGTTGTTAGTCGATAGAGGAAAGTGTCT	Fis - 3203|3204s-EBS2
BAP5967	ATGAAAGTCGACAAGGCGAACCTAAGTCCGC	Reverse primer for amplification of *pm1087*/*fis* operon
BAP5968	TTGCTTGGATCCTACAGGGAATCCAACCTAATC	Primer within *fis*
BAP5969	GCTTAAGGATCCGTATTATAGCGTCCCTTATCGG	Forward primer for amplification of *pm1087*/*fis* operon

a restriction sites are underlined.

**Figure 4:**
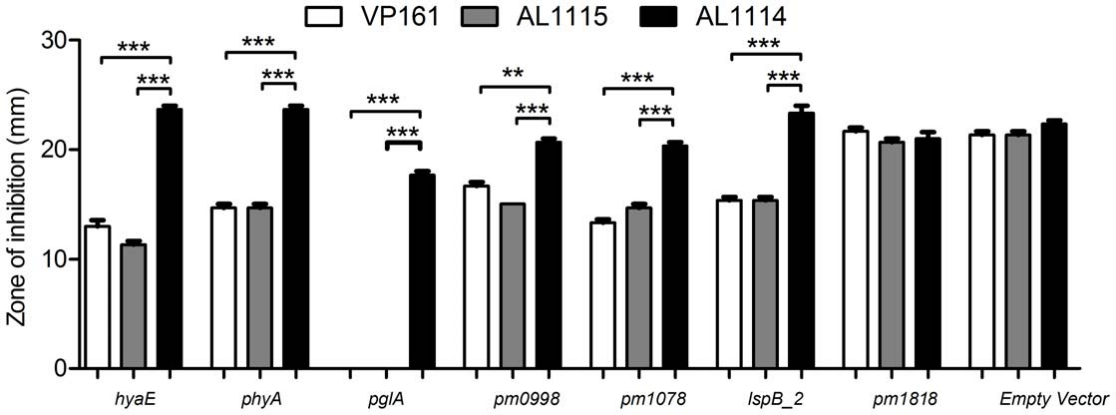
Sensitivity of *P. multocida* strains to kanamycin as determined by disc diffusion assays. The putative promoter-containing regions upstream of *hyaE*, *phyA*, *pglA*, *pm0998*, *pm1078*, *lspB_2*, and *pm1818* were cloned into the *P. multocida* promoter detecting vector pMKΩ, and the ability of the recombinant constructs (pAL597, pAL596, pAL795, pAL796, pAL797, pAL798 and pAL799 respectively) to confer kanamycin resistance to strains AL1114 (spontaneous acapsular strain), AL1115 (paired capsulated strain) and VP161 (capsulated wild-type parent) assessed. Empty pMKΩ vector was used as a negative control. The average relative expression was determined from three biological replicates and the values shown are the mean ±1 SEM. *** p<0.001, ** p<0.01.

To determine the activity of the *phyA* and *hyaE* promoters in both encapsulated and non-encapsulated strains, the intergenic region between *phyA* and *hyaE* (Fig. 2) was cloned in both orientations into the *P. multocida* promoter-detecting vector pMKΩ (Table 1; [24]) to generate pAL596 and pAL597. In pAL597 the pMKΩ kanamycin resistance gene is under the control of the *hyaE* promoter while in pAL596 it is under the control of the putative *phyA* promoter. These plasmids were then tested for their ability to confer kanamycin resistance on the wild-type *P. multocida* strain VP161, the encapsulated strain AL1115 and the acapsular variant AL1114 (Table 1). Both plasmids conferred higher levels of kanamycin resistance to the wild-type strain VP161 and the encapsulated strain AL1115 than when present in the acapsular strain AL1114 (Fig. 4). In contrast, the acapsular strain AL1114, harboring either pAL596 or pAL597 remained kanamycin sensitive, indicating that the *phyA* and *hyaE* promoters are inactive in this strain. These data show that both the *phyA* and *hyaE* promoters are active only when present in the encapsulated strains VP161 and AL1115, indicating that the activity of these promoters is regulated by a trans-acting transcriptional regulator which is inactive in the acapsular strain AL1114.

### Identification of the transcriptional regulator of *P. multocida* capsule expression

In order to identify the trans-acting transcriptional regulator responsible for the regulation of capsule expression, we sequenced the entire genomes of the acapsular variant AL1114 and its paired capsular strain AL1115, using high-throughput short read sequencing. Reference assemblies were generated for both strains using the fully annotated *P. multocida* PM70 genome [25] as a scaffold. These assemblies were then used to determine nucleotide differences between AL1114 and AL1115, and thus identify changes unique to AL1114. Three nucleotide changes were identified as unique to AL1114 when compared to AL1115 (Table 3); of these, the mutations within *asd* and *bioA* were silent and did not result in amino acid substitutions. However, the observed nucleotide T to C transition within an ORF annotated as encoding Fis, would result in a non-conservative L28S amino acid substitution within the Fis protein. The deduced *P. multocida* Fis protein is 99 amino acids in length and shares 80% identity with the 98 amino acid *E. coli* Fis (NCBI GeneID 947697; http://www.ncbi.nlm.nih.gov/sites/entrez?db=gene&cmd=Retrieve&dopt=Graphics&list_uids=947697) which has been shown to be a nucleoid-associated transcriptional regulator.

**Table 3:** Sequence differences identified by whole-genome Illumina short read sequencing between the acapsular strain AL1114 and the paired capsulated strain AL1115.

Pm70 Reference position^a^	Reference base	Base call (frequency) AL1115	Base call (frequency) AL1114	Gene (strand)	Resulting change in AL1114	P value
1282559	A	A (180), T (1)	G (230)	*fis* (−)	L28S substitution	1×10^−70^
1843077	A	A (138), G (12)	G (180)	*asd* (−)	silent	7×10^−35^
2140869	C	C (361), A (1)	C (171), A (21)	*bioA* (+)	silent	0.003

a All sequencing reads were aligned to the annotated *P. multocida* Pm70 genome sequence and the position of each mutation in relation to this sequence shown.

To confirm that the mutation observed in *fis* was associated with loss of capsule expression, the nucleotide sequence of *fis* was determined by Sanger dideoxy sequencing in all three acapsular variants (AL1114, AL1162 and AL1396) and their paired capsulated strains (AL1115, AL1163 and AL1397). Analysis of these sequence data confirmed the L28S mutation in Fis in AL1114 compared to AL1115. In AL1162, a G to T transversion at nucleotide 3 resulted in a change from the methionine start codon to the non-start codon isoleucine (ATT), stopping translation of Fis. In AL1396, a C to T substitution at nucleotide 222 resulted in a nonsense mutation (Q75*) and termination of the Fis protein at amino acid 74. These data are consistent with the hypothesis that mutation of *fis* was correlated with loss of capsule expression in all three spontaneous acapsular *P. multocida* strains.

### Functional Fis is required for capsule expression

In order to confirm Fis as the transcriptional regulator of capsule expression, we complemented each of the spontaneous acapsular variants with an intact copy of Fis *in trans*. In *E. coli*, *fis* is auto-regulated, and is expressed as part of a two gene operon together with the upstream gene *yhdG*, predicted to encode a tRNA-dihydrouridine synthase [14],[16],[26]; the same organization is observed in *P. multocida*. We initially attempted to clone *fis* by itself but were unable to successfully make this construct. Therefore, we cloned *fis*, the overlapping upstream gene *pm1087* and the predicted native promoter, into the *P. multocida* shuttle vector pPBA1100, generating the plasmid pAL727 (Table 1). The plasmid pAL727 and the vector only (pPBA1100) were separately introduced into each of the acapsular variants AL1114, AL1162 and AL1396 and quantitative HA assays performed to determine levels of capsule expression. In all cases, HA production was significantly higher in strains harboring pAL727 (AL1399, AL1401 and AL1403) than in control strains harboring empty vector (AL1398, AL1400 and AL1402) (p<0.05), although full complementation to wild type levels was not observed (Fig. 1).

### Inactivation of *fis* results in decreased capsule production

To confirm that Fis is essential for capsule production in *P. multocida* we constructed two independent, marker free *fis* mutations in the wild-type *P. multocida* VP161 strain using the intron-mediated TargeTron system (Sigma). We constructed two *P. multocida*-specific TargeTron vectors that were retargeted for the insertional inactivation of *fis* at nucleotide 48 (pAL706 and pAL708; Table 1). *P. multocida* transformants with intron insertions in *fis* were identified by PCR and, following curing of the mutagenesis plasmid, correct insertion of the intron in *fis* was confirmed by direct genomic sequencing (data not shown). A single insertion mutant generated from transformation with either pAL706 or pAL708 was selected for further study, and designated AL1404 and AL1405 respectively. Quantitative HA assays confirmed that insertional inactivation of *fis* resulted in the loss of capsular polysaccharide production in both AL1404 and AL1405 (Fig. 1; Table 1), showing unequivocally that Fis is essential for the production of capsular polysaccharide in *P. multocida* strain VP161.

### Identification of *P. multocida* genes regulated by Fis

Fis is known to be a global regulator in a number of bacterial species [15],[20],[27]. We therefore used whole genome DNA microarrays to compare the transcriptome of the acapsular variant AL1114 (expressing Fis L28S) with the encapsulated paired strain AL1115 (expressing wild-type Fis). Custom Combimatrix 12k microarrays were designed and used as described in Materials and Methods. Thirty one genes were identified as significantly down-regulated and eleven genes as significantly up-regulated in the acapsular variant AL1114 (Table 4). Seven of the ten capsule biosynthesis genes were identified as down-regulated in AL1114 compared to AL1115, supporting the qRT-PCR data showing reduced transcription of *phyA* and *hyaE*. Expression of the known *P. multocida* virulence gene *pfhB_2* and its predicted secretion partner *lspB_2* was reduced 3- to 4-fold in AL1114. In addition, the gene encoding the outer membrane lipoprotein PlpE, which is a surface exposed lipoprotein that can stimulate cross-serotype protective immunity against *P. multocida* infection [28], was also down-regulated in the acapsular strain AL1114. Other down-regulated genes included *pglA*, encoding a cryptic heparosan synthase; *exbB*, an iron-regulated virulence factor; *pm1042*, encoding a predicted LPS-specific phosphoethanolamine transferase; *htrA*, encoding a heat shock protein; *fruR*, encoding the fructose operon repressor; *glmS* encoding a fructose-6-phosphate aminotransferase, and a range of genes encoding proteins of unknown function. Eleven genes were identified as up-regulated in the acapsular strain. These included *pm1819* and *pm1820*, which encode proteins with similarity to the *Salmonella* SrfB and SrfC proteins, both putative virulence factors also controlled by Fis [23], *tadC*, a gene predicted to be involved in an outer membrane secretion system, and seven genes encoding proteins of unknown function. Several of the genes identified as differentially expressed were physically linked on the chromosome and given their similar expression patterns we predict that these are expressed as operons (e.g., *plpE*, *pm1516* and *pm1515*; *pm0996*, *pm0997* and *pm0998*; *pm1819*, *pm1820* and *pm1821*; Table 4).

**Table 4:** Genes identified as differentially regulated between the *P. multocida* wild-type strain and a *fis* mutant strain as determined by DNA microarray analysis.

GeneID^a^	Locus	Predicted function	COG^b^	Fold Change (log2)	Adjusted p Value
*Genes down-regulated in AL1114*					
1244121	*hyaE*	Capsule Biosynthesis	D	−2.52	0.001
1244123	*hyaC*	Capsule Biosynthesis	M	−1.81	0.001
1244127	*hexB*	Capsule Biosynthesis	G/M	−1.55^c^	0.008
1244125	*hexD*	Capsule Biosynthesis	M	−1.49^c^	0.003
1244126	*hexC*	Capsule Biosynthesis	M	−1.47	0.004
1244120	*phyA*	Capsule Biosynthesis	M	−1.36^c^	0.004
1244122	*hyaD*	Capsule Biosynthesis	M	−1.04^c^	0.024
1244345	PM0998	Unknown	M	−3.13	0.001
1244344	*PM0997*	Putative membrane protein	M	−1.24	0.004
1244343	*PM0996*	Putative ABC transporter	V	−1.07	0.009
1244863	*PM1516*	Putative DNA methylase	L	−1.95^c^	0.001
1244864	*plpE*	Protective lipoprotein	-	−1.94	0.001
1244862	*PM1515*	Unknown	-	−1.15	0.030
1243405	*lspB_2*	Hemolysin accessory protein	U	−1.98	0.001
1243406	*pfhB_2*	Filamantous haemagluttinin	S	−1.57	0.003
1245078	*glmS*	Fructose-6-phosphate aminotransferase	M	−1.94^c^	0.004
1244389	*PM1042*	Putative PEtn transferase	R	−1.94^c^	0.001
N/A	Unknown	Unknown VP161 specific ORF fragment	-	−1.69	0.003
1243932	*sohB*	Putative protease	O/U	−1.49^c^	0.022
1243761	*pglA*	Cryptic heparosan synthase	M	−1.41^c^	0.010
1245040	*cysK*	Cysteine synthase	E	−1.40^c^	0.043
1244425	*PM1078*	Hemin binding receptor	P	−1.40^c^	0.004
1243799	*PM0452*	Hypothetical periplasmic protein	P	−1.36^c^	0.050
1244215	*fruR*	Fructose repressor	K	−1.36^c^	0.004
1244081	*htrA*	Heat shock protein	O	−1.32^c^	0.010
1244442	*PM1095*	Unknown	-	−1.32^c^	0.031
1244533	*exbB*	Iron regulated virulence protein	U	−1.24^c^	0.023
1244083	*dnaK*	Predicted chaperone	K	−1.15^c^	0.043
1244505	*PM1158*	Putative methyltransferase	H	−1.11^c^	0.024
1244397	*pm1050*	Unknown	M	−1.06^c^	0.033
1244287	*nrdD*	Ribonucleoside-triphosphate reductase	F	−0.79^c^	0.035
*Genes upregulated in AL1114*					
1245166	*PM1819*	*srfB*, putative virulence factor	S	0.92^c^	0.012
1245167	*PM1820*	*srfC*, putative virulence factor	S	1.04^c^	0.013
1245168	*PM1821*	Unknown	-	1.68^c^	0.003
1244564	*PM1217*	Unknown	-	1.48	0.044
1244194	*tadC*	Hypothetical protein	N/U	1.09^c^	0.034
1244021	*PM0674*	Unknown	-	1.85^c^	0.008
1244889	*pckA*	Predicted phosphoenolpyruvate carboxykinase	C	2.13^c^	0.044
1243866	*PM0519*	Unknown	S	2.18	0.044
1245193	*ptsB*	Sucrose-specific PTSII	G	2.19	0.047
1244890	*PM1543*	Unknown	-	2.39^c^	0.007
1244799	*PM1452*	Unknown	S	2.70^c^	0.024

a NCBI accession number of the identified gene.

b Cluster of Orthologous Groups functional group (http://www.ncbi.nlm.nih.gov/COG/).

c Only one of two probes for this gene was significantly differentially regulated.

### Confirmation of the microarray data

In order to confirm the differential expression of some of the genes identified by the microarray analyses, two different methods were used. Firstly, the putative promoter regions of each of the down-regulated genes *pglA*, *pm0998*, *pm1078, lspB_2* and the region upstream of *pm1818* containing the putative promoter for the operon containing the up-regulated genes *pm1819, pm1820* and *pm1821*, were cloned into the *P. multocida* promoter probe vector pMKΩ (Table 1), generating the recombinant plasmids pAL795, pAL796, pAL797, pAL798 and pAL799, respectively (Table 1). The recombinant plasmids were transformed into the acapsular strain AL1114 (expressing Fis L28S), the capsulated paired strain AL1115 (expressing wild-type Fis) and the wild-type parent strain VP161 (Table 1). With the exception of pAL799, each of the recombinant pMKΩ derivatives conferred higher levels of kanamycin resistance to AL1115 and the wild-type VP161 than to the acapsular variant AL1114 (Fig. 4). These data support the microarray results and show that the activity of the promoters for *pglA*, *pm0998*, *pm1078* and *lspB_2* are significantly reduced in the absence of wild-type Fis. Each of the strains harboring pAL799 showed similar levels of kanamycin resistance regardless of the capsule phenotype, suggesting that the cloned fragment in this construct does not contain a Fis regulated promoter, or that the fragment does not contain all the necessary Fis binding sites required for repression of this promoter.

**Figure 5:**
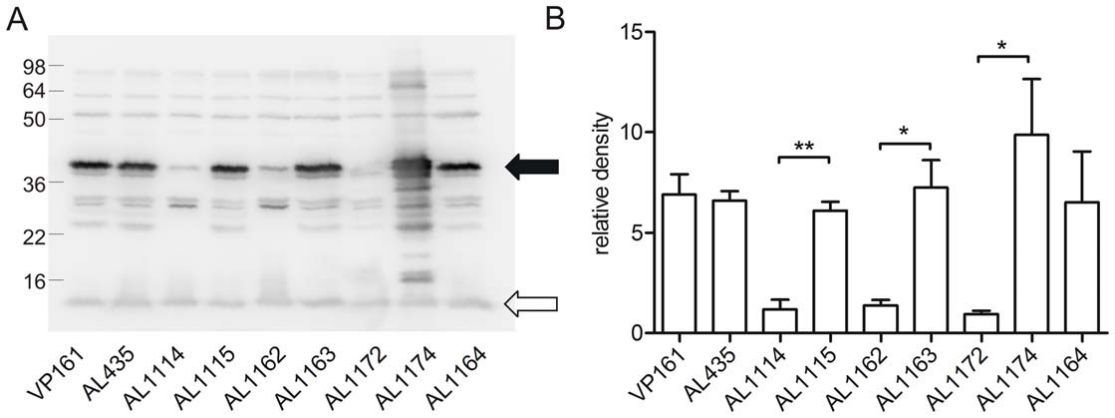
Western immunoblot and densitometry analysis of *P. multocida* PlpE expression in acapsular, capsulated and control strains. Western immunoblot using chicken antiserum against recombinant PlpE (A) and the corresponding quantitive densitometry analysis (B). The levels of PlpE expression were measured in the capsulated parent strains (VP161, AL435), the acapsular *fis* mutants (AL1114, AL1162) and paired capsulated strains (AL1115, AL1163), a single-crossover *plpE* mutant (AL1172; Hatfaludi, Al-Hasani and Adler, unpublished data), complemented *plpE* mutant (AL1174) and the acapsular *phyB* mutant strain that produces no HA (AL1164). The filled arrow head represents the position of PlpE, whilst the unfilled arrowhead represents the background band used as a loading control for densitometry normalization. Each bar represents the average normalized densitometry readings from triplicate biological replicates ±1 SEM. ** p<0.01, * p<0.05.

As a second method of confirmation, qRT-PCR and western immunoblot analyses were undertaken to confirm the reduced expression of *plpE* in AL1114. PlpE is a predicted outer membrane lipoprotein which can stimulate cross-serotype protective immunity against *P. multocida* [28]. Microarray analysis indicated that the *plpE* transcription was reduced by approximately 3.8-fold in the spontaneously arising acapsular strain (Table 4). Transcriptional analysis of *plpE* by qRT-PCR confirmed that the transcription of this gene was significantly reduced (data not shown). Western immunoblots using antiserum generated against recombinant PlpE (Fig. 5) confirmed that PlpE expression was significantly reduced in the spontaneously arising acapsular variants AL1114 and AL1162, as well as a directed *plpE* mutant AL1172 (Table 1), but not in the directed *phyB* acapsular strain AL1164. Therefore, PlpE mRNA levels are positively regulated by Fis.

## Discussion

The expression of a polysaccharide capsule is critical for the virulence of *P. multocida* [8],[9]. While there is evidence that the level of capsule expression in *P. multocida* responds to certain environmental conditions (such as growth in the presence of antibiotics, low iron or specific iron sources such as hemoglobin [29]–[32], there is no information on the mechanism of capsule regulation. There have been numerous reports of spontaneous loss of capsule expression in *P. multocida* strains following *in vitro* passage [10],[11], but the mechanism by which this occurs has not been determined. Previous work with laboratory-derived, spontaneous acapsular strains has indicated that loss of capsule expression was associated with reduced transcription of genes within the capsule biosynthesis locus [12]. In this study, we identified three independent spontaneous acapsular strains and showed that loss of capsule expression in these strains was also due to reduced transcription of the *cap* locus. Furthermore, we showed that this reduced transcription was due to point mutations within the gene encoding the global transcriptional regulator Fis. Thus, Fis is essential for capsule expression and these experiments define a mechanism by which spontaneous capsule loss can occur and identify for the first time a transcriptional regulator required for capsule expression in *P. multocida*. Importantly, while Fis has been shown to be involved in the regulation of a large number of genes in a range of bacterial species, to our knowledge this is the first report showing a role for Fis in the regulation of capsule biosynthesis. Furthermore, this is the first report that a functional Fis protein is expressed in *P. multocida* and that it acts as a regulator of gene expression.

Fis was initially identified as the factor for DNA inversion in the Hin and Gin family of DNA recombinases [33],[34]. Subsequently, diverse roles for Fis have been described, including both positive and negative regulation of gene expression. Fis has also been identified in other members of the Pasteurellaceae, including *Haemophilus influenzae* Rd. While the *H. influenzae* Fis shared 81% identity with the *E. coli* Fis, it did not display identical activity [35]. Interestingly, *P. multocida* Fis shares 92% identity with the *H. influenzae* Fis but only 80% identity with *E. coli* Fis.

Structurally, Fis folds into four α-helices (A–D) and a β-hairpin [36]. Helices A and B provide the contacts between Fis monomers, facilitating dimer formation, whereas the C and D helices form a helix-turn-helix motif that is essential for DNA binding [37]–[39]. We identified three spontaneous acapsular strains with point mutations within *fis*. The Fis mutation L28S is predicted to result in a highly unstable protein, as a mutation in the equivalent position in the *E. coli* Fis (L27R) resulted in an unstable protein with no discernible activity [36]. However, as the *P. multocida* Fis shares only 80% amino acid identity with the *E. coli* Fis, we can not rule out the possibility that the *P. multocida* L28S Fis is a partially functional protein that is impaired in only some specific functions of WT Fis. Computational models also suggest a requirement for hydrophobic residues at this position [40] and the substitution of leucine by the hydrophilic residue serine would significantly reduce the hydrophobicity at this position. The second spontaneous acapsular variant, AL1162, contained a mutation within the *fis* start codon, which would result in complete abrogation of Fis expression. Finally, the *fis* gene in AL1396 contained a nonsense mutation at nucleotide 222, terminating protein translation at amino acid 74. This mutant Fis protein would lack the last 16 amino acids, including the helix-turn-helix motif that is essential for DNA binding [39].

As Fis is known to regulate a number of genes in other species, we used DNA microarrays to compare the transcriptome of wild-type *P. multocida* and the Fis L28S mutant (AL1114) during exponential growth. Comparison of the *fis* mutant strain with wild-type *P. multocida* identified 31 genes (representing at least 20 predicted operons) as positively regulated by Fis, and 11 genes (representing at least nine operons) as negatively regulated by Fis. In *E. coli*, two transcriptional studies have been conducted comparing gene expression in wild-type and Fis mutant strains. The first study identified more than 200 genes that were regulated by Fis at various growth phases (>2 fold change, p<0.05) [15]. The second study identified >900 genes (21% of the *E. coli* genome) that were significantly differentially regulated in the *fis* mutant (false discovery rate <1%), although only 17 of these were >2 fold differentially regulated [27]. Interestingly, approximately 70% of the 900 genes shown to be differentially regulated in the second study showed no Fis binding as determined by chromatin immunoprecipitation coupled with high resolution whole genome-tiling arrays [27]. In *P. multocida* we identified 42 genes as differentially regulated by Fis during the exponential growth phase. During this growth phase the regulation of *P. multocida* genes by Fis was skewed towards the up-regulation of genes by Fis (70% of operons), indicating that Fis generally acts as a transcriptional activator, a finding consistent with previous studies [15],[27].

Both *P. multocida* and *E. coli* Fis share significant similarity across the C-terminal DNA binding region, suggesting that they may recognise similar sequences. However, using the available *E. coli* position specific weight matrix [15], we were unable to identify conserved sites upstream of all of the Fis regulated genes identified in the DNA microarray experiments. This is not entirely unexpected, as Fis has been shown to bind a variety of AT-rich sequences more or less non-specifically [13]. Indeed, the ability of Fis to induce DNA bending is probably the most important factor in its ability to control transcription [41]. The relatively low number of *P. multocida* genes regulated by Fis in the exponential growth phase is somewhat surprising. However, as Fis expression in other bacteria has been shown to be growth phase dependant [15],[42],[43], it is likely that other *P. multocida* genes will be differentially regulated by Fis during different growth phases. While an equivalent L27R mutation in *E. coli* Fis results in an unstable protein, we can not rule out the possibility that the L28S mutation in *P. multocida* Fis results in a protein that retains some, but not all, Fis regulatory functions. Thus, it is possible that more genes might be observed as differentially regulated in a Fis null mutant strain and we are currently investigating this possibility.

Of the 42 differentially expressed genes identified in the *P. multocida* Fis mutant, a large number (16/42; 38%) encode proteins that are surface expressed or involved in the synthesis of surface exposed structures. Clearly Fis regulates the genes involved in the biosynthesis of, and surface presentation of, capsular polysaccharide, and expression of these genes is critical for virulence. Fis is also involved in the regulation of the surface exposed virulence factor PfhB_2 and its outer membrane secretion partner LspB_2 and the surface expressed lipoprotein PlpE. Both PlpE and PfhB2 can provide protection against *P. multocida* infection when used as vaccine antigens [28],[44]. Other surface-associated Fis-regulated genes identified by the DNA microarray analyses included: *pm1042*, encoding a transferase predicted to attach phosphoethanolamine to lipid A of LPS; *tadC*, a gene located within the tight adherence locus that has been shown in *Aggregatibacter actinomycetemcomitans* to be responsible for non-specific attachment to surfaces and is required for full expression of the RcpABC outer membrane proteins [45]; *pm1078*, encoding a putative iron-specific ABC transporter component; and the genes encoding PM0998 [46], PM1050 and PM1819 [47] which have all been experimentally shown to be present in outer membrane sub-fractions of the *P. multocida* proteome. Fis also regulates the expression of ExbB, an inner membrane protein that interacts with TonB and is critical for iron uptake and virulence in *P. multocida* [48].

The *P. multocida* filamentous hemagglutinin PfhB_2 has been previously identified as a virulence factor [49],[50] and recent work has shown that vaccination with recombinant PfhB_2 can induce protective immunity in turkeys [44]. In other species such as *Bordetella pertussis* the secretion of the filamentous hemagglutinin (FHA) into the extracellular medium is reliant on the outer membrane protein FhaC [51]. In both *Haemophilus* and *Bordetella*, expression of the outer membrane secretion component is controlled by the two-component signal transduction systems *cpxRA* [52] and *bvgAS* [53] respectively. It is clear from our work that in *P. multocida* Fis co-ordinately activates the expression of PfhB_2 and its upstream predicted secretion partner LspB_2. However, we can not exclude the possibility that expression of these genes is also dependent on other regulatory mechanisms such as a two component signal transduction system.

Of particular significance is the finding that Fis regulates *plpE*, which encodes the cross protective antigen PlpE [28]. Interestingly, previous studies on spontaneous acapsular strains have indicated that loss of a 39–40 kDa lipoprotein was correlated with the loss of capsular polysaccharide and it was hypothesized that the lack of expression of this protein on the surface was due to the physical loss of capsule [11], [54]–[56]. We propose that the lipoprotein identified in the above studies is in fact PlpE and its expression is reduced in spontaneous acapsular strains because of the transcriptional down-regulation of the gene due to the absence of Fis.

In summary, we have characterized three independently derived, spontaneous, acapsular variants of *P. multocida*. In all three strains, loss of capsule production was due to a single nucleotide change in the gene encoding the nucleoid-associated protein Fis, identifying for the first time a mechanism for spontaneous capsule loss and a regulator critical for *P. multocida* capsule expression. Furthermore, analysis of gene expression in the *fis* mutant provides convincing evidence for the role of Fis in the regulation of critical *P. multocida* virulence factors and surface exposed components.

## Materials and Methods

### Bacterial strains, plasmids, media and growth conditions

The bacterial strains and plasmids used in this study are listed in Table 1. *E. coli* was grown in 2YT broth and *P. multocida* in brain heart infusion (BHI) broth or nutrient broth (NB). Solid media were obtained by the addition of 1.5% agar. When required, the media were supplemented with streptomycin (50 µg/ml), spectinomycin (50 µg/ml), kanamycin (50 µg/ml) or tetracycline (2.5 µg/ml).

### Molecular techniques

Restriction digests and ligations were performed according to the manufacturers' instructions using enzymes obtained from NEB (Beverly, MA) or Roche Diagnostics GmbH (Mannheim, Germany). Plasmid DNA was prepared using Qiagen DNA miniprep spin columns (Qiagen, GmbH, Germany). *P. multocida* genomic DNA was isolated using the RBC genomic DNA purification kit (RBC, Taiwan). Amplification of DNA by PCR was performed using Taq DNA polymerase (Roche) and, when required, PCR amplified products were purified using the Qiagen PCR purification Kit (Qiagen, GmbH, Germany). Oligonucleotides used in this study (Table 2) were synthesized by Sigma, Australia. For transformation of plasmid DNA into *P. multocida*, electro-competent cells were prepared as described previously [8] and electroporated at 1800V, 600Ω, 25µF. DNA sequences obtained by Sanger sequencing were determined on an Applied Biosystems 3730S Genetic Analyser and sequence analysis was performed with Vector NTI Advance version 10 (Invitrogen, Carlsbad, CA).

### Generation of a single cross-over mutant in *hyaB*

For inactivation of *hyaB* in *P. multocida* strain VP161 we used the previously described single-crossover insertional mutagenesis method which utilizes the λ *pir*-dependent plasmid pUA826 [57]. A 720 bp internal fragment of *hyaB* was amplified by PCR using oligonucleotides BAP4399 and BAP4400 (Table 2), and cloned into the *Sal*I site of pUA826tpi, generating pAL499 (Table 1). This plasmid was then mobilized from *E. coli* SM10 λ *pir* into *P. multocida* strain AL435 by conjugation, and insertional mutants selected on BHI agar containing tetracycline, spectinomycin and streptomycin. Single cross-over insertion of the recombinant plasmid into *hyaB* was confirmed by PCR using either of the genomic flanking primers BAP276 or BAP169 (Table 1) together with BAP2782 located within the plasmid pUA826tpi. One transconjugant with the correct insertion in *hyaB* was selected for further study, and designated AL919 (Table 1).

### Disc diffusion assays

Disc diffusion assays were performed on soft agar overlays. Briefly, 100 µl of each *P. multocida* overnight culture was mixed with 3 ml of BHI containing 0.8% agar and immediately poured onto a BHI agar (1.5%) base. Kanamycin at a range of concentrations, was absorbed onto sterile Whatman paper discs, placed on the agar overlay containing *P. multocida*, and incubated for 18 h at 37°C. Inhibition of growth was determined as the diameter of the zone of clearing around the discs.

### Quantitative HA assays

Crude capsular material was extracted as described previously [58] with the following modifications. One ml of a *P. multocida* overnight culture was pelleted by centrifugation at 13 000 *g*, and washed once with 1 ml of PBS. Washed cells were resuspended in 1 ml of fresh PBS, incubated at 42°C for 1 h, then pelleted, and the supernatant, containing crude capsular polysaccharide, collected. HA content was determined as described previously [8].

### Fluorescent primer extension

Fluorescent primer extension was performed as described previously [59], with the following modifications. First strand cDNA synthesis was performed with SuperScript III RT (Invitrogen) according to the manufacturer's instructions. A typical reaction contained 10 µg of total RNA, 1mM dNTPs and 6-FAM labeled primer at a final concentration of 100 nM. Fragment length analysis of FAM-labeled cDNAs was performed by the Australian Genome Research Facility (AGRF, Melbourne). cDNA fragments were separated on an AB3730 DNA analyzer (Applied Biosystems), and sizes determined using Genemapper V3.7 software (Applied Biosystems).

### High-throughput genome sequencing and analysis

High-throughput sequencing was performed on an Illumina GA2 (Illumina, USA) by the Micromon Sequencing Facility (Monash University, Australia). Two lanes of 36-bp single end data were generated for both AL1114 and AL1115. Raw sequence data from both strains was aligned independently to the *P. multocida* PM70 genome sequence using SHRiMP [60] (average read depth ∼200), which is able to produce alignments in the presence of single nucleotide polymorphisms (SNPs) and insertions and deletions (indels). These alignments were then used to compile, for each position in the reference, a contingency table of counts of observed bases in each of the two samples, and the significance of each different base call was determined using Fisher's exact test. The determined significance values were corrected for multiple testing using the Bonferonni adjustment. Raw read data were also assembled *de novo* using VELVET [61] or CLC genomics workbench (CLC).

### Complementation of spontaneous mutants and construction of plasmids for promoter analysis

For complementation of spontaneous acapsular strains, wild-type Fis and the overlapping upstream ORF *pm1087*, were amplified from *P. multocida* VP161 genomic DNA using oligonucleotides BAP5967 and BAP5969 (Table 2) containing *Sal*I and *Bam*HI sites respectively. The amplified fragment was digested and cloned into *Sal*I- and *Bam*HI-digested pPBA1100, generating pAL727 (Table 1). This plasmid was then used to transform the acapsular strains AL1114, AL1162 and AL1396, generating AL1399, AL1401 and AL1403. As a control, empty pPBA1100 was also used to transform each of these strains, generating AL1398, AL1400 and AL1402 (Table 1).

For analysis of promoter activity in *P. multocida*, predicted promoter containing fragments were amplified by PCR and cloned into the *P. multocida* promoter detecting vector pMKΩ, which contains a promoterless kanamycin resistance gene (Table 1, [24]). The genomic region containing the *hyaE* promoter and the predicted *phyA* promoter was amplified by PCR using the oligonucleotides BAP5091 and BAP5092 (Table 2) and cloned in both orientations into pMKΩ to generate pAL596 and pAL597. The predicted promoter containing fragments upstream of the genes *pglA*, *pm0998*, *pm1078*, *lspB_2* and *pm1818* (the first gene in a putative operon containing *pm1819*, *pm1820* and *pm1821*) were amplified by PCR using the oligonucleotide pairs BAP5834 and BAP5835 (*pglA*), BAP5828 and BAP5829 (*pm0998*), BAP5826 and BAP5827 (*pm1078*), BAP5832 and BAP5833 (*lspB_2*) and BAP5838 and BAP5839 (*pm1818*) (Table 2) and each fragment cloned into pMKΩ to generate pAL795, pAL796, pAL797, pAL798 and pAL799, respectively (Table 1). Each of the recombinant plasmids was then transformed into the wild-type parent strain VP161, the acapsular *fis* mutant strain AL1114 and its paired capsulated derivative AL1115. Promoter activity from the cloned fragment in pMKΩ was assessed semi-quantitatively by disc diffusion assays where a reduced zone of growth inhibition around kanamycin impregnated discs indicated a higher level of kanamycin resistance and therefore promoter activity from the cloned fragment.

### Construction of *P. multocida* directed *fis* mutants

The *E. coli*/*P. multocida* shuttle vector pAL99 (Table 1) was used to generate two TargeTron vectors, pAL692 and pAL705, for the generation of marker-free *fis* mutants in *P. multocida*. The spectinomycin/streptomycin resistant TargeTron vector pAL692 (Table 1) was constructed as follows: pAL99 was amplified by PCR using outward facing primers that flank the *aph3* gene (BAP5358 and BAP5359) and digested with *Eco*RV. This fragment was ligated to an *Eco*RV-digested PCR fragment containing the *aadA* gene amplified from pUA826 using the primers BAP5360 and BAP5361. Following ligation the plasmid was digested with *Hin*dIII and *Fsp*I and ligated to a 4 kb *Hin*dIII/*Fsp*I-digested fragment of pJIR750ai encoding the TargeTron intron and *ltrA* gene (Table 1) such that transcription would be driven by the constitutive *P. multocida tpiA* promoter. For construction of the kanamycin resistant TargeTron vector pAL705 (Table 1), the *aph3* gene was amplified from pAL99 using the primers BAP5433 and BAP5434 then cloned into *Eco*RV-digested pAL692, thereby replacing the *aadA* gene.

Retargeting of the intron within each vector to nucleotide 48 of *fis* was performed as per the TargeTron user manual (Sigma) using the oligonucleotides BAP5932-BAP5934 (Table 1). The retargeted mutagenesis plasmids, pAL706 (Kan^R^) and pAL708 (Strep^R^/Spec^R^) (Table 1) were used to transform *P. multocida* VP161 and antibiotic resistant transformants selected on either spectinomycin or kanamycin. Insertion of the intron into the *P. multocida* genome was detected using the *fis*-specific oligonucleotides BAP5967 and BAP5968 (Table 1); the presence of the intron resulted in a 0.9 kb increase in the size of the PCR product compared to the PCR product generated from the wild-type *fis* (data not shown). Mutants confirmed by PCR to have insertions in *fis* were cured of the replicating TargeTron plasmid by a single overnight growth in NB broth without antibiotic selection, followed by patching of single colonies for either Strep^S^Spec^S^ or Kan^S^. Strains cured of replicating plasmid were confirmed as *fis* mutants by additional PCR amplifications to show the presence of the intron and the absence of a copy of wild-type *fis* (data not shown). Finally, *fis* mutations were confirmed by direct genomic sequencing using intron-specific primers.

### RNA isolation and quantitative real-time RT-PCR (qRT-PCR)

Bacteria were harvested from triplicate BHI cultures at late log phase (∼5×10^9^ CFU/ml) by centrifugation at 13 000 *g*, and RNA was isolated using TRIzol reagent (Gibco/BRL) as described by the manufacturer. The purified total RNA was treated with DNase (15 U for 10 min at 37°C), and then further purified on RNeasy mini columns (Qiagen). Primers for qRT-PCR were designed using the Primer Express software (ABI) (BAP4995-BAP4998; Table 2). RT reactions were routinely performed in 20 µl volumes, containing 5 µg total RNA, 15 ng random hexamers, 0.5mM dNTPs and 300 U SuperScript III Reverse Transcriptase (Invitrogen) at 42°C for 2.5 h. The synthesized cDNA samples were diluted 50-fold prior to qRT-PCR, which was performed using an Eppendorf realplex^4^ mastercycler with product accumulation quantified by incorporation of the fluorescent dye SYBR Green. Samples were assayed in triplicate using 2 µl of diluted cDNA with SYBR Green PCR master mix (ABI) and 50 nM concentrations of each gene-specific primer. The concentration of template in each reaction was determined by comparison with a gene-specific standard curve constructed from known concentrations of *P. multocida* strain VP161 genomic DNA. *gyrB* was used as the normalizer for all reactions. All RT-PCRs amplified a single product as determined by melting curve analysis.

### Microarray analysis

Custom Combimatrix 12k microarrays (Combimatrix, USA) were designed based on the published sequence of *P. multocida* PM70 [25], with the addition of probes specific for ORFs previously identified as unique to *P. multocida* strain VP161 [62]. cDNA for microarray hybridizations was prepared as for qRT-PCR, except that RNA contamination was removed from the cDNA by the addition of NaOH followed by column purification (Qiagen minElute, Qiagen). A total of 2 µg of purified cDNA was labeled using KREAtech Cy3-ULS (KREAtech, The Netherlands), and used in hybridizations with the Combimatrix 12k microarrays as per the manufacturer's instructions. Triplicate hybridized arrays were scanned on a Genepix 4000b scanner, and spot intensities determined using Microarray Imager v5.9.3 (Combimatrix, USA). After scanning, each array was immediately stripped and re-scanned as per manufacturers' instructions. Spot intensities of stripped arrays were used as background correction for the quantification of subsequent hybridizations. Spots from duplicate probes were averaged, and the averaged probe intensities analyzed using the LIMMA software package [63] as follows. Background correction was performed using the LIMMA “normexp” method [64], and the Log_2_ values calculated. Between-array quantile normalization [65] was then applied to the log transformed spot intensities. A moderated t-test on the normalized log intensities was performed to identify differentially expressed genes. Probes were sorted by significance, and the False Discovery Rate (FDR) [66] used to control for multiple testing. Probes showing ≥2-fold intensity change between AL1114 and AL1115, with a FDR of <0.05 were considered differentially expressed (Table 2). Two probes were designed for all genes over 500 bp in length; genes were classed as differentially expressed if one or both probes showed a differential expression of ≥2- fold. DNA microarray experiments were carried out according to MIAME guidelines and the complete experimental data can be obtained online from the NCBI Gene Expression Omnibus (http://www.ncbi.nlm.nih.gov/geo/) submission number GSE17686.

### SDS-PAGE, western immunoblot and densitometry

SDS-PAGE was performed as described previously [67]. Proteins separated by SDS-PAGE were transferred to Immobilon-P membranes (Millipore). Western immunoblot analysis was performed using standard techniques [68], with chicken anti-recombinant PlpE as the primary antibody and peroxidase-conjugated anti-chicken immunoglobulin (raised in donkeys) as the secondary antibody. Blots were visualized using CDP-Star (Roche), and imaged on a Fujifilm LAS-3000 chemiluminescent imager (Fujifilm). Densitometry was performed using Multi-gauge software v2.3 (Fujifilm)
